# Research Tools to Investigate Movements, Migrations, and Life History of Sturgeons (Acipenseridae), with an Emphasis on Marine-Oriented Populations

**DOI:** 10.1371/journal.pone.0071552

**Published:** 2013-08-22

**Authors:** Troy C. Nelson, Phaedra Doukakis, Steven T. Lindley, Andrea D. Schreier, Joseph E. Hightower, Larry R. Hildebrand, Rebecca E. Whitlock, Molly A. H. Webb

**Affiliations:** 1 Fraser River Sturgeon Conservation Society, Vancouver, British Columbia, Canada; 2 Scripps Institution of Oceanography, University of California San Diego, San Diego, California, United States of America; 3 National Marine Fisheries Service, Southwest Fisheries Science Center, Santa Cruz, California, United States of America; 4 Department of Animal Science, University of California Davis, Davis, California, United States of America; 5 U. S. Geological Survey, NC Cooperative Fish and Wildlife Research Unit, Department of Applied Ecology, NC State University, Raleigh, North Carolina, United States of America; 6 Golder Associates Ltd., Castlegar, British Columbia, Canada; 7 Stanford University, Hopkins Marine Station, Pacific Grove, California, United States of America; 8 U. S. Fish and Wildlife Service, Bozeman Fish Technology Center, Bozeman, Montana, United States of America; Aristotle University of Thessaloniki, Greece

## Abstract

Worldwide, sturgeons (Acipenseridae) are among the most endangered fishes due to habitat degradation, overfishing, and inherent life history characteristics (long life span, late maturation, and infrequent spawning). As most sturgeons are anadromous, a considerable portion of their life history occurs in estuarine and marine environments where they may encounter unique threats (e.g., interception in non-target fisheries). Of the 16 marine-oriented species, 12 are designated as Critically Endangered by the IUCN, and these include species commercially harvested. We review important research tools and techniques (tagging, electronic tagging, genetics, microchemistry, observatory) and discuss the comparative utility of these techniques to investigate movements, migrations, and life-history characteristics of sturgeons. Examples are provided regarding what the applications have revealed regarding movement and migration and how this information can be used for conservation and management. Through studies that include Gulf (*Acipenser oxyrinchus desotoi*) and Green Sturgeon (*A. medirostris*), we illustrate what is known about well-studied species and then explore lesser-studied species. A more complete picture of migration is available for North American sturgeon species, while European and Asian species, which are among the most endangered sturgeons, are less understood. We put forth recommendations that encourage the support of stewardship initiatives to build awareness and provide key information for population assessment and monitoring.

## Introduction

**Figure 1:**
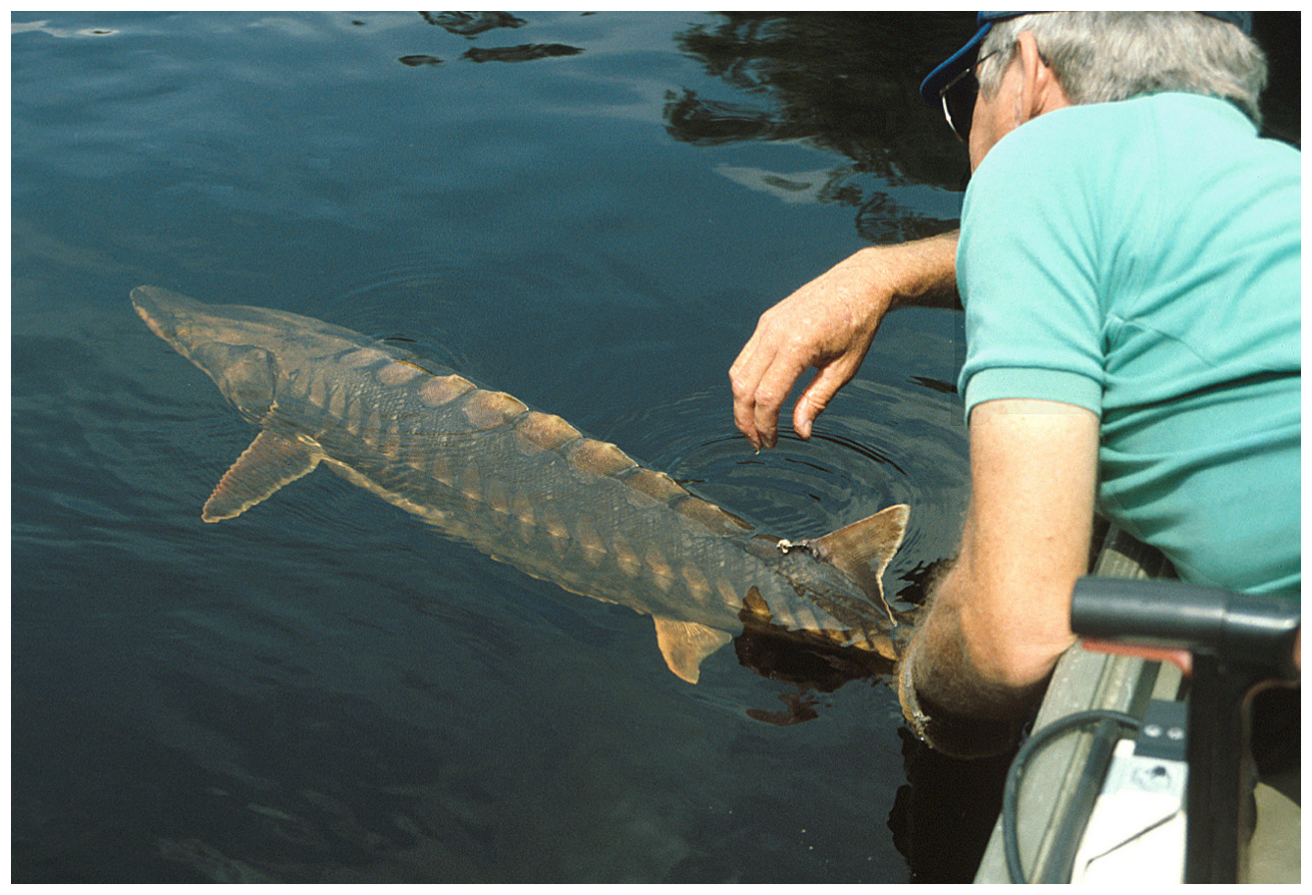
Gulf Sturgeon (*Acipenser oxyrinchus desotoi*). *Photo: Joe Hightower*.

There are 27 species of sturgeons and paddlefishes (Order Acipenseriformes) in rivers, lakes, estuaries, near-shore oceanic environments, and inland seas across the northern hemisphere (Table 1). The two species of paddlefishes (Family Polyodontidae) are strictly freshwater in life history while the 25 sturgeon (Family Acipenseridae) species include 16 species that enter into estuaries, oceans, or seas during some part of their life cycle. Sturgeons have five rows of bony scutes and snouts with sensory barbels (Figure 1). All sturgeons spawn in freshwater habitats. The 16 marine-oriented species (species that spend a significant portion of their life history in marine environments) occur on all of the continents to which sturgeons are endemic, including North America, Europe, and Asia.

**Table 1:** Sturgeons (Family Acipenseridae) of the world, their marine distributions, and their respective IUCN* Category.

Species	Distribution	IUCN * Category
*Acipenser baerii,* Siberian Sturgeon	FW, E/Bays	Endangered
*Acipe*n*ser brevirostrum,* Shortnose Sturgeon	FW, E, Occasionally Coastal	Vulnerable
*Acipenser dabryanus,* Yangtze Sturgeon	FW	Critically Endangered (pe)
*Acipenser fulvescens,* Lake Sturgeon	FW	Least Concern
*Acipenser gueldenstaedtii,* Russian Sturgeon	E, Sea (Black, Caspian, Azov)	Critically Endangered
*Acipenser medirostris,* Green Sturgeon	E, Coastal (Baja, California to the Bering Sea)	Near Threatened
*Acipenser mikadoi,* Sakhalin Sturgeon	E, Coastal (Bering Sea, Sea of Okhotsk, Sea of Japan, south Sakhalin Island; range very restricted at present); $$	Critically Endangered
*Acipenser naccarii,* Adriatic Sturgeon	E, Coastal; Adriatic Sea; range very restricted at present; $$	Critically Endangered (pe)
*Acipenser nudiventris,* Ship Sturgeon	E, Sea (Black, Caspian, Azov)	Critically Endangered
*Acipenser oxyrinchus Acipenser oxyrinchus desotoi, Gulf Sturgeon Acipenser oxyrinchus oxyrinchus,* Atlantic Sturgeon	E, Coastal (Gulf of Mexico to Quebec)	Near Threatened
*Acipenser persicus,* Persian Sturgeon	E, Sea (Black, Caspian)	Critically Endangered
*Acipenser ruthenus,* Sterlet	FW	Vulnerable
*Acipenser schrenckii,* Amur Sturgeon	E, Coastal (distribution uncertain); $$	Critically Endangered
*Acipenser sinensis,* Chinese Sturgeon	E, Coastal (Yellow and East China seas; range very restricted at present)	Critically Endangered
*Acipenser stellatus,* Stellate Sturgeon	E, Sea (Black, Caspian, Azov)	Critically Endangered
*Acipenser sturio,* European (Baltic) Sturgeon	E, Coastal (Baltic Sea; once throughout Western Europe; range very restricted at present)	Critically Endangered (pe)
*Acipenser transmontanus,* White Sturgeon	E, Coastal (Aleutian Islands to Monterey California)	Least Concern
*Huso huso,* Beluga Sturgeon	E, Sea (Black, Caspian, Azov)	Critically Endangered
*Huso dauricus,* Kaluga Sturgeon	E. Coastal (Sea of Okhotsk, Tatar Strait, Sea of Japan); $$	Critically Endangered
*Scaphirhynchus albus,* Pallid Sturgeon	FW	Endangered
*Scaphirhynchus platorynchus,* Shovelnose Sturgeon	FW	Vulnerable
*Scaphirhynchus suttkusi,* Alabama Sturgeon	FW	Critically Endangered
*Pseudoscaphirhynchus hermanni,* Dwarf Sturgeon	FW	Critically Endangered
*Pseudoscaphirhynchus fedtschenkoi,* Syr Darya Sturgeon	FW	Critically Endangered (pe)
*Pseudoscaphirhynchus kaufmanni,* Amu Darya Sturgeon	FW	Critically Endangered

* International Union for Conservation of Nature.

NOTES: FW  =  primarily freshwater over most of life history.

E  =  estuarine.

Sea  =  inhabits seas but not oceans.

$$  =  additional research is required to determine the marine distribution of these species.

pe  =  possibly extinct in the wild.

Sources from **REFERENCES**: [10], [61], [211]–[213].

Sturgeons are generally long-lived fishes that exhibit late onset of maturity, slow growth, and infrequent reproduction [1]. Some species have life spans of well over 100 years, and sexual maturity may be attained as late as 20–25 years of age or more in females [2]. These life-history characteristics are linked to low maximum rates of population growth and underlie intrinsic vulnerability to exploitation-induced decline and a low recovery capacity [3], [4]. Damming of rivers can be particularly detrimental to many sturgeon populations as it can reduce or eliminate spawning and egg/larvae incubation habitats and change important environmental cues relating to flow regimes and hydrographic characteristics [5]. Human activities such as dredging, channelization, dyking, bank stabilization, in-river construction, and shoreline development can affect important juvenile rearing habitats [6]. Other prominent impacts to sturgeon include hybridization, pollution, introduced species, reduced food supply, water diversions, and saltwater intrusion (into important spawning and rearing habitats) [1], [7]. Their value as the source of black caviar (the unfertilized roe of female sturgeon) has led sturgeons (particularly in the Black and Caspian seas) to be the target of intensive legal and illegal fisheries [1], [2]. This has resulted in the collapse of several species and stocks of sturgeon [1]. The combination of inherent life-history challenges, habitat degradation, and exploitation has reduced sturgeon abundance to such low levels that the sturgeons are now considered one of the most endangered groups of animals in the world according to the International Union for Conservation of Nature (IUCN). In 2010, the IUCN reviewed the status of the 25 species (and two subspecies) of sturgeon (Acipenseridae) in their “Red List” (list of threatened species); four species are listed as “threatened,” five species as “endangered,” and 18 species are “critically endangered” (likely to become extinct in a generation). The IUCN ranking approach is conservative; for example, the IUCN classifies White Sturgeon (all populations) as “least concern,” whereas White Sturgeon in Canada are classified “endangered” under the national ranking system [8], and in the US, the Kootenai River White Sturgeon is federally listed as “endangered.”

Sturgeons are also some of the least-well-known of the major taxa of concern in terms of spatial distribution and abundance, particularly for oceanic and nearshore phases of their life history. Almost all of the sturgeons that enter into saltwater have been understudied with respect to where they go and why (Table 1) and many endangered species have received relatively little scientific attention and study [9]. Researching these information gaps is extremely important for conservation of habitats and distinct populations. Here, we explore these issues while providing case studies and tools for examining movement and distribution of these ancient fishes.

### 1.1. Rational for Research: Understanding Movement and Migration – and why it Matters

Sturgeons may move from one location or habitat type to another to feed, reproduce, or overwinter. River movement can be complex and include multi-step migrations [10] and include movement between and among rivers suggesting a meta-population structure [11]. Within a species, populations can differ on the timing of migrations into river systems, time spent within the river (holding), and the distance (upstream) from the marine environment where spawning occurs. Directed migrations from overwintering locations to feeding habitats, in some cases timed to intercept specific, localized prey species, may be a population-based behaviour; other populations of the same species may exhibit an entirely different food-related migration pattern based on the abundance/timing of localized prey. Protecting the genetic heritage and diversity of a species requires an understanding of these complexities at both the species and population levels, and an understanding of specific life-stage habitat requirements. Species and populations subject to harvest typically benefit from well-managed harvest regimes that incorporate respective migration information. There may be sex-specific differences in the timing of movements (often males arrive at the spawning grounds before females) so harvest/interception regimes may need to be sensitive to this in order to maintain the male:female sex-ratio balance within the population.

Preserving the evolutionary potential of a species and its ability to respond to environmental change requires understanding the number of distinct populations within a species. Natal homing fidelity is thought to be strong and thus in many cases rivers, or sections of a watershed between natural barriers, often define populations. On the other hand, metapopulations may also exist [11]. If individual populations differ in terms of abundance and reproductive capacity, researchers and fishery managers may want to minimize mortality of individuals from certain river systems while they are in marine environments. Movement studies can provide the necessary information at both population and species levels and can provide the basis for protective schemes under national and international legislation. Characterizing environmental parameters that correlate with behaviour is further useful in evaluating the potential impact of habitat alteration (e.g. changes to the annual hydrograph of a sturgeon-bearing river). In some areas, introduction of non-native sturgeons and hybridization between species (e.g. Scaphirhynchus) due to habitat alteration and/or unintentional release from aquaculture (e.g. in Europe) makes understanding the movement and life history of introduced sturgeons or hybrid populations an important conservation consideration [12], [13].

Knowledge about movement and the spatial distribution of populations can also improve population assessments and help in specifying suitable management or recovery plans. Population assessments are still lacking for most species and are often hindered by an insufficient understanding of spatial distribution. Sturgeon species and/or populations with an anadromous life-history strategy may require assessment models that account for the seasonal movement patterns of different life-history stages exploited by fisheries [14], [15]. Tagging data can be incorporated into spatially explicit models to allow more accurate estimation of fishing mortality rates experienced by different age classes where fishing intensity varies by area [14], [16].

Insufficient data for parameterisation of models for migratory species with complex life histories can lead to large uncertainties in resource assessment and the likely effects of alternative management actions. The lack of data for key parameters of population dynamics has been identified as a contributor to the depleted and endangered status of many sturgeon populations throughout their range [17], [18]. Below, we present recent studies of the Gulf and Green Sturgeon to show how the information gained from these representative studies has been used to direct subsequent activities toward species conservation and the protection of key habitats.

### 1.2. Case Studies: The Well-studied Gulf and Green Sturgeon

#### 1.2.1. Gulf Sturgeon

**Figure 2:**
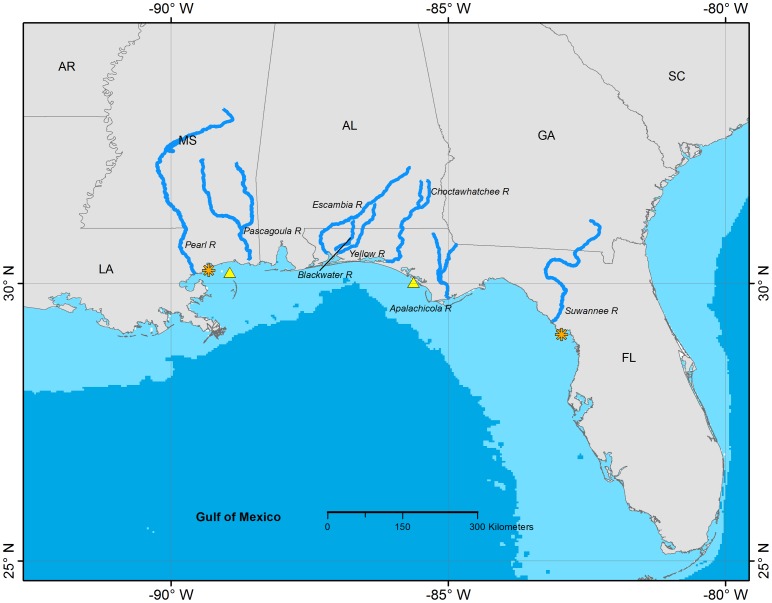
Documented distribution of Gulf Sturgeon in North America, determined from acoustic and archival telemetry projects. The orange asterisks mark the easternmost and westernmost locations of confirmed detections of acoustic-tagged Gulf Sturgeon. Gulf Sturgeon spawn in coastal rivers including the eight shown on this map. Spawning and non-spawning Gulf Sturgeon typically remain in coastal rivers until fall and occupy estuarine and nearshore marine waters during winter. Yellow triangles indicate winter concentration areas for Gulf Sturgeon from two or more river systems. The 100 m isobath is shown as the light blue areas near the coast.

The anadromous Gulf Sturgeon, *Acipenser oxyrinchus desotoi*, a subspecies of the Atlantic Sturgeon, *Acipenser oxyrinchus*, occurs in most Gulf of Mexico river systems from the mouth of the Mississippi River to the west coast of Florida (Figure 2). Both mature and immature Gulf Sturgeon undergo freshwater migrations, typically entering coastal rivers in March or April and outmigrating to the ocean in September or October [19]. The cool-water period of estuarine or marine residency is critical for growth and reproduction, as Gulf Sturgeon do not feed during their freshwater residency.

Early information about Gulf Sturgeon distribution and migration came primarily from commercial fishing [20]. Fishing operations in individual rivers were mostly short-lived (due to overharvest and subsequent fishery closures, as is typical for many in-river sturgeon fisheries), but the catches did provide some insights into the timing and extent of migration. More detailed information came from surveys in the 1980s conducted in response to declining catches and the species' listing by the state of Florida as “threatened” [21], [22]. Marking with conventional tags mostly showed the range and extent of migration (e.g. recaptures of tagged fish by anglers below a dam or by commercial shrimp trawlers in Gulf of Mexico waters), whereas the first use of electronic tags (and manual/mobile tracking) provided insights about holding, staging, and spawning areas [22]; the use of radio tags in this study allowed the authors to characterize occupied riverine habitats in the Apalachicola River and to relate the timing of upstream and downstream migrations to environmental cues.

Radio transmitters worked well for manual tracking of Gulf Sturgeon over long distances in rivers, but these transmitters cannot be detected in brackish or marine water. In the late 1980s a telemetry study was conducted wherein Gulf Sturgeon were tagged with both radio and sonic tags [23]. This was also the first Gulf Sturgeon study to use stationary receivers to detect and log passage events (in this case, to detect sonic tags as fish moved through barrier island passes [23]).

The listing of Gulf Sturgeon in 1991 as a threatened species under the U. S. Endangered Species Act provided a further boost to research activity. For example, radio tracking studies in the Choctawhatchee River were initiated to identify potential spawning sites, with confirmation through the use of artificial substrates to collect the adhesive eggs of Gulf Sturgeon [24]. Deployment of artificial substrates in a grid design provided fine-scale information about spawning habitat in the Suwannee River [25]. Marine habitat studies using sonic tags (and more recently, archival temperature-logging and pop-up archival tags) showed that Gulf Sturgeon sometimes moved long distances along the shoreline and primarily used shallow nearshore areas [26]–[29]. The fish occupying these marine habitats were often from multiple river systems; for example, [27] reported that Gulf Sturgeon from the Yellow, Choctawhatchee, and Apalachicola rivers were located within a 25-km stretch of coastline (eastern winter concentration area shown on Figure 2). The co-occurrence of Gulf Sturgeon from the Pearl and Pascagoula rivers has been documented [29] in the concentration area off Mississippi (western area shown on Figure 2). Thus, marine and estuarine threats and management efforts may affect more than one population.

Genetic studies have also aided in understanding Gulf Sturgeon migration patterns. For example, it has been shown that within a basin, genetic structure exists at least at the drainage level and possibly at the level of tributary rivers within the basin [30]. The genetic analyses were helpful in interpreting telemetry results since some fish were tagged outside their natal drainage and others were captured or detected in multiple drainages.

These research results formed the basis for the Gulf Sturgeon recovery plan and led to the designation of critical habitats. These important habitats included upper-basin spawning sites with limestone bluffs and outcroppings, estuarine and marine feeding sites with preferred substrates and benthic fauna, and summer resting areas. Genetic results showed strong natal river fidelity, so critical habitat was defined in each of the seven river systems containing currently reproducing populations (Pearl, Pascagoula, Escambia, Yellow/Blackwater, Choctawhatchee, Apalachicola, and Suwannee). This resulted in designation of nearly 2,800 river km as critical habitat for conservation of the species.

#### 1.2.2. Green Sturgeon

**Figure 3:**
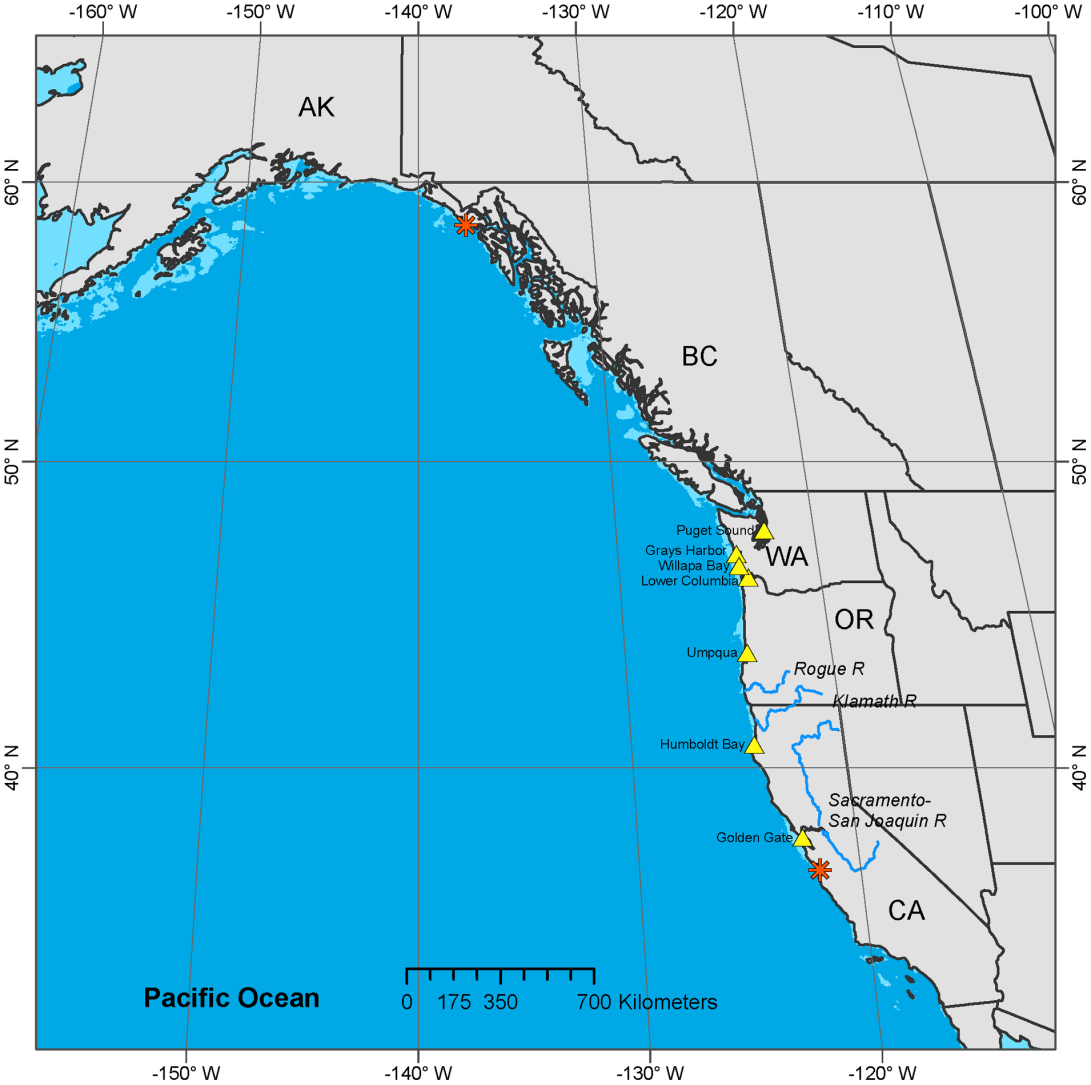
Documented distribution of Green Sturgeon in North America, determined from acoustic telemetry project with fixed receiver array. The orange asterisks mark the northernmost and southernmost locations of confirmed detections of acoustic-tagged Green Sturgeon. Green Sturgeon spawn in California in the Sacramento and Klamath rivers, and in Oregon in the Rogue River (shown in blue). They spend summers in estuaries and bays in California, Oregon, and Washington, and utilize the coastal ocean between southern Alaska and Baja California, Mexico, generally remaining in water less than 100 m deep. Summer aggregation areas are shown as yellow triangles. The 100 m isobath is shown as the light blue areas near the coast.

In contrast to the relatively well-studied Gulf Sturgeon, the North American Green Sturgeon was little studied until 2002, when the US National Marine Fisheries Service received a petition to list it under the US Endangered Species Act. A severe lack of demographic and basic life-history information hampered the subsequent status review [31]. A particularly troubling unknown was the population origin(s) of Green Sturgeon that form dense aggregations in certain estuaries during summer months. Green Sturgeon were known to use just three rivers for spawning (the Sacramento and Klamath rivers in California, and the Rogue River in Oregon), and to spend much of their lives in marine waters between Alaska and Baja California (Figure 3). The purpose of the summertime estuarine aggregations was unknown, as was the proportion of Green Sturgeon exhibiting this aggregation behaviour. Green Sturgeon in these aggregations are vulnerable to capture in gillnet fisheries that target White Sturgeon and Pacific salmon species, and face environmental threats from activities associated with shellfish aquaculture and industrial activities in the estuaries where they aggregate.

The recent development of new telemetric tagging systems made it feasible to rapidly close some of these information gaps. Initial work focused on Green Sturgeon in the Rogue River, using radio and acoustic tags to learn that Green Sturgeon migrate into rivers in the early spring for spawning in up-river areas, and then hold in deep pools over the summer prior to emigration in the fall when flows rise with the onset of the rainy season [32]. Tagged sturgeon returned to the river to spawn every two to four years [33]. Rogue River fish were also tagged with pop-off archival tags (PAT), which revealed that they remain in fairly shallow water (50–80 m) when in the coastal ocean, and showed that they migrate north to the west coast of Vancouver Island in the fall [34].

A broader study using acoustic tags showed that Green Sturgeon make extensive seasonal migrations among spawning areas, over-summering in various estuaries and bays, and over-wintering areas in the coastal ocean, with many individuals using areas around northern Vancouver Island [35], [36]. Further PAT work, using longer tag deployments, also showed this seasonal migration pattern, and fairly constrained depth and temperature distributions during the winter. Acoustic tags also revealed extensive use of and movement among non-natal estuaries. Green Sturgeon from different populations mixed together in common estuaries, but at different rates. Natal estuaries were used almost exclusively by fish from the associated natal river [36]. Green Sturgeon were also shown to have diverse patterns of migration within and among populations. Within the Sacramento River, acoustic tags revealed that a seasonal water diversion dam was a serious impediment to the spawning migration of Green Sturgeon [37]. Habitat data associated with tag detections was used to gain further insight into freshwater [38] and fine− [39] and coarse-scale [40] marine habitat preferences and seasonal patterns of distribution. Captive rearing of Green Sturgeon is providing important information on salinity tolerance and the timing for successful transition to marine waters, as well as optimal temperature for egg hatching, embryogenesis, and larval and juvenile survival [41]–[46].

In summary, the application of electronic tag technology to study Green Sturgeon revealed novel, reliable, and useful/relevant information regarding marine migrations and habitat use, spawning locations and periodicity, and both marine and freshwater life history attributes. While tagging revealed clear overall migration patterns, it also revealed a diversity of migratory behaviours that are likely important to consider in the conservation and protection of this species. Complementary genetic studies have determined that two distinct population segments exist within Green Sturgeon that correspond to northern and southern populations, and that estuarine aggregations contain individuals from multiple populations [47]. Data acquired from electronic tagging has already been the basis of many of the conservation regulations put in place after the listing of the southern distinct population segment of Green Sturgeon by the US federal government. Critical habitat for Green Sturgeon was designated in 2009 along the continental shelf of the US from southeast Alaska to central California out to a depth of 110 m, as well as in a number of estuaries and bays in Washington, Oregon, and California. Regulatory biologists are using the acoustic tagging data in their assessments of permit applications for activities such as dredging and dredge spoil disposal, large construction projects within and along the shores of estuaries, and the management of groundfish trawl fisheries in the coastal ocean.

### 1.3. Case Studies: Less-studied Species in Regions of Europe and Asia

#### 1.3.1. Caspian Sea

The Caspian Sea is one of the most important areas for sturgeons globally and has supplied much of the world's wild caviar. Five of the species that inhabit this region (Stellate, Beluga, Persian, Russian and Ship Sturgeon; Table 1), including those that produce the highly prized Beluga, Sevruga and Osetra caviar, are anadromous. Adult sturgeons move into river systems to reproduce in spring and sometimes winter and fall. Most adults that migrate into rivers in spring are thought to leave the river just after spawning, but those that migrate in the fall and winter may overwinter. The patterns of spawning migrations of Caspian Sea sturgeons differ amongst species, and the damming of rivers has likely altered migration patterns [48].

After hatching, larvae and fingerlings remain within the rivers for several months depending on the species: for some species (e.g. Stellate and Russian), fingerlings may overwinter in river deltas while for others (e.g. Beluga), fingerlings will migrate into the Caspian Sea for rearing. Information regarding the behaviour and movement of juveniles as well as adults within rivers has been gathered through fisheries-dependant surveys and fisheries-independent studies using nets. While a few tagging studies have been attempted (e.g. [49]), none have been conducted on the scale necessary to provide species-specific population and movement/migration information. Such studies would be of further benefit in understanding the reproductive periodicity of the different species.

It is unclear whether adult sturgeon in the Caspian Sea return to their natal rivers for reproduction. Genetic studies using mitochondrial DNA have not shown differentiation amongst river populations [50], but preliminary data from microsatellites suggest that natal homing does occur. Hatchery supplementation and stocking in rivers may be affecting the natural population structure that once existed because hatcheries do not always use broodstock collected in the river system where the hatchery-produced juveniles are released. Detections of genetic haplotypes from the non-native Siberian Sturgeon in tissue samples taken from Caspian Sea Russian Sturgeon have raised questions regarding hatchery practices, non-native releases, and the life history of this potential hybrid (see section 2.4). There is a great need to understand the species and population structure of Caspian Sea sturgeons so that management activities reflect and protect the various populations.

Within the Caspian Sea environment, comprehensive information about the distribution of sturgeons is lacking [48]. Much of what is known has been collected through trawl surveys, which have been limited spatially and geographically. The vertical distribution of sturgeons in the Caspian has been examined through trawl and drift net surveys, but these studies have been similarly limited. Since at-sea trawl survey information is used to generate abundance estimates and harvest quotas, a better understanding of distribution and movement is important. Tagging and tracking studies would greatly complement available data from the trawl and net surveys (see [48] for compiled information), allowing better fisheries management and design of protected areas around feeding and spawning areas.

#### 1.3.2. Western Europe Outside of the Black Sea

In Western Europe, sturgeons have been heavily affected by historic fishing and habitat alteration as well as non-native release (see section 2.4); the presence of exotic species and hybrids is particularly problematic in this region. The area is represented by small, sometimes relict populations such as the European (Baltic) Sturgeon *(A. sturio)* and Adriatic Sturgeon (*A. naccarii*; Table 1). For these critically endangered species, the emphasis is on recovery and restoration; distribution and movement data are extremely useful for identifying critical and important habitats.

Restoration projects for European and Atlantic Sturgeon have released marked (mostly tagged with external T-bar tags) juveniles from captive breeding facilities to track subsequent movements and habitat use [51]. Acoustic telemetry has been used to study young European Sturgeon in the Gironde River estuary and the influence of tidal cycles on their movement [52]. [53] used acoustic telemetry to monitor reintroduction trials for American Atlantic Sturgeon (*A. oxyrinchus oxrinchus*) to the Baltic Sea and European Sturgeon to the Elbe River in Germany, providing key information on food selectivity, migration patterns, and potential sources of mortality. This study was important to the success of reintroduction efforts (2,000–5,000 tagged fish released per year) and in guiding planned restoration and habitat protection efforts. Tagging will eventually reveal whether reintroduced animals survive to adulthood and return to their rivers of release to reproduce. This study as well as [54] and [55] not only provide knowledge for species restoration but also insight on the migratory and habitat use patterns of juvenile sturgeon, the least studied life history stage in terms of movement and migration. Rearing of European Sturgeon in captivity also provides information on life history parameters associated with light, temperature, and salinity that are useful for the purpose of inferring population preferences and requirements in the wild [56], [57].

#### 1.3.3. Black Sea

The Black Sea is home to the same commercially important species of sturgeon as found in the Caspian Sea, with the Danube River supporting the largest populations. Sturgeons in this region have also been impacted by fishing and habitat degradation and the focus of scientific research is often on restoration, recovery, and management. In the Black Sea, acoustic tagging studies have been used to examine spawning location and timing for Stellate (A. stellatus) and Russian Sturgeon (A. gueldenstaedtii) in the lower Danube River [58]. A high rate of interception of tagged fish as indicated by reported fishery returns (38% in 1998 and 28% in 1999) limited the success of the effort. Stellate and Russian Sturgeon appeared particularly sensitive to capture and handling, with over 50% of tagged fish subsequently aborting their respective upstream migrations. Tagging is again being attempted in the Black Sea, as is the use of a DIDSON acoustic camera (see section 2.6.2), to investigate movements [59]. The current tagging study may be more successful than the previous effort due to a moratorium on sturgeon fishing currently in place in the Romanian and Bulgarian portions of the Danube River. New research that incorporates microchemistry techniques is focusing on the use of trace elements to understand migratory patterns [60]. Future monitoring and assessment studies that incorporate tagging will be important in understanding whether fishing moratoria are being respected and if sturgeon populations are successfully recruiting and rebounding.

#### 1.3.4. Russian Far East and China

The Russian Far East and China include little-known (and under-studied) anadromous sturgeon species [61], [62]. The Amur (*A. schrenckii*) and Kaluga (*Huso dauricus*) Sturgeon of the Amur River are commercially exploited for meat and caviar. These species are thought to have complex population structures, undertaking considerable migrations within the river (Amur Sturgeon), and marine environment (Kaluga). The Sakhalin Sturgeon inhabits the Russian Far East and is believed to have a primarily marine life history; it is one of the least studied of the sturgeons and is also one of the most critically endangered, with spawning populations possibly reduced to a single river. The Chinese Sturgeon, *A. sinensis*, has benefited from studies aimed at understanding movements in relation to dams and habitat protection [63]–[65]. As with the Caspian and European species, these species would benefit from studies of migration, movement, and distribution, so that appropriate recovery and management plans can be produced.

## Research Techniques

### 2.1. Tagging and Marking

The application of individually numbered or coded tags to fish prior to release is typically referred to as “tagging.” The term “marking,” while sometimes used as a substitute for tagging, is typically used to reference batch or group “mark” applications. Subsequent recaptures of tagged fish can yield information on movement, time at large, growth, etc., whereas recaptures of marked fish will provide general information only regarding the entire “mark group.” If recapture and reporting rates are sufficiently high, capture-recapture data from tagging studies are very useful for population assessment, providing information on population size, size/age structure, movement rates, fishing/natural mortality, and fish behaviour.

#### 2.1.1. Considerations

Sturgeon are long-lived animals; thus, tags or marks that can be positively identified many years following release are ideal. Although not always feasible, determination of sex and maturational stage of individual sturgeon used in long-term tagging studies can provide greater insights regarding interpretation of subsequent movement and migration patterns. If tagging involves tag implants in the body cavity (typically electronic tags), the process should include visual determination of sex and stage or analysis of plasma concentrations to identify sex [66], [67]; see section 2.1.4 of this paper.

The use of uniquely identifiable tags can provide comparative growth and condition information for long-term studies with adequate sample sizes [68]. Length data (i.e., fork length) collected during initial tag release events and again during subsequent recaptures can be used to calculate daily growth rates for individual fish (based on the number of days at large between the release and subsequent recapture events). Daily growth can be expanded to provide estimates of annual growth, which in turn can be pooled and averaged for size/age groups of fish; these comparative data can provide insights regarding changes in overall population growth rates and condition over time [68].

#### 2.1.2. Marking

Marking sturgeon with traditional methods such as dye marking, tattoos, pigment implantation, or freeze branding is not recommended for studies that intend to report recaptured marks over a long time span (i.e., several years), as these marks cannot be clearly identified on sturgeon within several months or a year of application. Another more-permanent marking approach to be considered, with caution, is scute removal [69], [70]. Scutes are one of the few body parts of sturgeon that can be removed that will not grow back. A combination of scutes removed from pre-determined locations on the sturgeon can translate into information such as location, year, and month. Since scute removal may induce high levels of stress, it is not recommended for use on sturgeon populations that are critically endangered.

Other permanent marks that have been applied to sturgeon include barbel clipping and fin ray removal. Barbel removal is not recommended due to the importance of barbels to sturgeon sensory physiology and the reduction in fitness and increase in mortality it can cause [71]. The total or partial removal of the lead pectoral fin ray has gained popularity and utility for sturgeon aging (fin ray cross sectioning) and can also be used for genetic analyses (fin tissue) and microchemistry analyses (fin ray composition analyses). The removal or clipping of a fin ray can also constitute a short-term (less than 5 years) mark, typically as a secondary mark to a tag application. However, care should be taken to clip the fin ray beyond the articulated base (above the insertion point with the body wall) or mortality rates could increase [72]. As with scute removal, fin ray removal may elevate levels of stress and reduce swimming performance (short term) and is not recommended for use on critically endangered populations.

#### 2.1.3. Conventional Tagging

Tag types for sturgeon fall into three major categories: external tags, internal tags, and electronic (telemetry) tags. For the purposes of this paper, internal tags will be limited to Passive Integrated Transponder (PIT) tags, which are small, individually coded tags that are injected (with a specialized tool) into the body musculature or internal body cavity of sturgeon and are detected with a hand-held electronic tag reader. The use of electronic tags (that can be attached externally or internally) and utility of associated telemetric tag types (radio, acoustic, satellite) is presented in section 2.1.4 of this paper.

**External tags** – External, non-telemetry tags are typically attached to dorsal or lateral locations on the sturgeon with the intention to minimize the impact or influence of the tag (encourage natural behaviour) and maximize tag retention. Some external tags are applied with an application tool (such as “anchor” or “T” tags, that are applied with a “tagging gun”), while others may be attached by hand, sometimes with the use of an applicator or needle. Tag materials include plastic, PVC, nylon, and metal, and may be available in various colors to attract attention and/or assist with individual or batch identification. External tags are typically labelled with information that includes a unique tag number, and may include contact information for tag reporting, such as an address or phone number. Retention rates for external tags applied to sturgeon vary; some can maintain retentions above 70% for up to 3 years while other external tag application/types may not provide the high levels of tag retention rates required for mark-recapture analyses [69]. T-bar anchor tags can have especially good retention rates [73], [74].

Common external tags used for sturgeon include: the “monel” tag (metal tag that is usually clamped around the front or back of the dorsal fin, operculum, or pectoral fin); the “disc” tag (a flat round plastic tag with a small hole in the center, typically attached to the base of the dorsal fin with stainless wire); and the “anchor” or “T” tag, which is typically a 3–5 cm length of small-diameter PVC tube with a “T” shaped plastic anchor at one end. The “cinch” tag (also known as a “loop” tag) is similar to a long anchor tag except that the ends are attached (forming a loop). Popular attachment points for external tags are typically near the dorsal fin (either anterior or posterior of the fin, alongside the fin, or through the base of the fin) or the edge of the operculum or pectoral fin (popular with monel or similar metal “clamp” tags).

**Figure 4:**
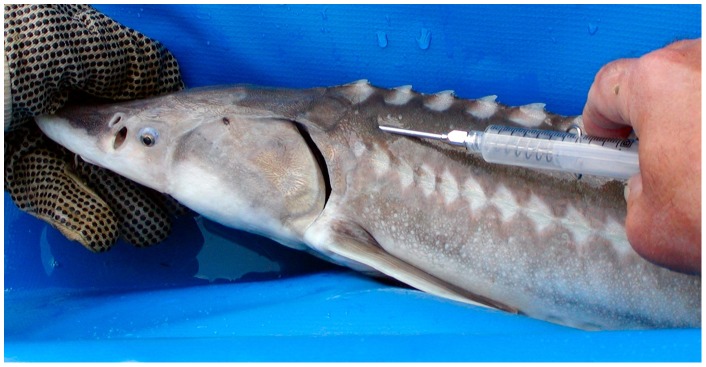
Illustration of the preferred location of PIT tag application on a juvenile White Sturgeon. The PIT tag is injected just beneath the skin, about 1 cm behind the head plate, on the left side of the dorsal scute line. *Photo: Fraser River Sturgeon Conservation Society.*

**Internal (PIT) tags** – A popular and effective tag for sturgeon is the PIT tag, a small, uniquely coded electronic tag that is applied internally via a hand-held applicator (syringe). Long-term retention rates for PIT tags are typically above 95% [69], [73], [75]. Popular PIT tag insertion locations are in the body cavity, base of pectoral fin or dorsal fin (or between base of dorsal fin and lateral line), and behind head plate (left or right of dorsal line). The position behind the head plate (Figure 4) has gained popularity in recent years due to lower tag loss rates and also based on concerns regarding potential human consumption of tagged sturgeon (tags in the “head” area are less likely to be consumed [68]). PIT tags used for sturgeon are typically 2 mm in diameter and 10–14 mm in length. Following application, there is no visible external indication that the fish has been tagged; the fish must be “scanned” with a hand-held electronic PIT tag reader (scanner) to determine if the fish has been tagged. PIT tag readers are typically battery powered, and display the tag number on a small screen. Tags can be detected with most hand-held tag readers from a distance up to about 20 cm, and the signal can be detected through water, flesh, etc.

PIT tags can also be detected with PIT tag antennas that are built into passive underwater apparatus such as tubes, loops, or mats; when a PIT tagged sturgeon passes through or near the antenna, the tag code is logged with time and date in the memory of a receiving unit (connected by cable to the antenna apparatus or array) that is later downloaded. This application may be effective to gather recapture data for constricted locations where tagged sturgeon are forced to pass through a confined area. For some applications, remote PIT tag detection may provide information to calculate overall survival [76]; however, without a secondary detection strategy to document the passage of non-tagged sturgeon (such as a video camera, or test sampling) to determine mark rates, the tag data alone will not allow sufficient data to produce abundance estimates.

***Considerations for PIT tags*** – PIT tags are typically more expensive (in the order of 5–10 times more expensive) per tag than most external tag types but have high retention rates. PIT tag information is transmitted from the tag by way of a specific radio frequency; both the tag and tag reader must be compatible and using the same frequency. Thus, when committing to a long-term research project that uses PIT tags, it is important to determine the long-term availability of both the tags and the compatible tag readers. Also, researchers should determine if other sturgeon researchers that have worked or are working on the target population or species have applied PIT tags to sturgeon. If so, new studies that include PIT tag applications may want to consider using the same tag frequency so that previously tagged sturgeon can be identified.

#### 2.1.4. Telemetry Tagging

The rapid advancement of electronic tag technology (telemetry) has facilitated an explosion of research on sturgeon life history, which in turn has generated novel insights into migratory behaviour, habitat use, and demographic processes. Electronic tagging offers advantages over traditional tagging approaches requiring large sample sizes to overcome low recapture rates. Electronic tags can be categorized by several characteristics. One distinction is whether tags transmit data in real-time to receivers (telemetry tags) or store data to internal memory for transmission at some later date (archival tag). Telemetry tags can be further differentiated by signal transmission mode: acoustic or radio. To date, telemetry tags have been most widely used in sturgeon research, although archival tags have been used with great success in studies of oceanic fish and marine birds.

Acoustic tags transmit identification codes (and potentially other data from pressure or temperature sensors) to hydrophones over a limited range (generally <1 km). Radio tags are similar, with differences related to signal transmission mode: radio waves propagate well through air and freshwater, but poorly in hard or saline water. Because acoustic or radio tagged fish need not be recaptured and can be detected at a distance, strategically placed receivers can collect large amounts of data on tagged fish. Combined acoustic and radio tags (CART) combine both transmission modes, allowing radio receivers to be used in freshwater and acoustic receivers to be used in brackish and saltwater. For both kinds of tags, information about the tagged fish is only collected from fish within the detection area of receivers. This shortcoming can be overcome by archival tags, which record signals from various sensors and store them internally. Typical information recorded by archival tags include pressure (depth), temperature, and light level, which allows estimation of location based on sea surface temperature and the time of sunrise and sunset [77]. Other sensors include electromyograms (EMG) that record muscle contractions, and accelerometers and tilt sensors that provide data on movement and orientation. Data from archival tags can be recovered by recapturing the tagged animal (and downloading data from the tag) or by telemetric transfer of archived data to satellites after pre-programmed release from the tagged animal.

**Use and utility of telemetry tags** – We searched the primary literature for sturgeon studies reporting the use of electronic tags, and found 55 relevant studies (summarized in Table 2). To date, the most frequent application of electronic tagging has been to study habitat use and movement of wild sturgeon within rivers. A substantial fraction of these studies have identified the timing and location of spawning, a topic of obvious interest to those working on conservation of sturgeon. These studies have relied on both acoustic and radio tracking, often in combination, and typically have made use of mobile tracking to detect tagged sturgeon. A related type of study examines the movement and habitat use of cultured sturgeon after release.

**Table 2:** Summary of recent sturgeon studies that utilized electronic tag technologies to acquire movement information, by species, electronic tag type, and tracking method.

Study Type	Species	Tag Type	Tracking Method	REFERENCE
**Habitat use and movements in rivers**	*A. brevirostrum*	AC, RA	MT	[214]
	*A. fulvescens*	AC	MT	[215]
	*A. fulvescens*	AC, RA	MT	[216]
	*A. fulvescens*	RA	MT	[217]
	*A. gueldenstaedtii*	AC	FR, MT	[58]
	*A. medirostris*	AC	FR	[187]
	*A. medirostris*	AC, RA	FR, MT	[33], [218]
	*A. medirostris*	RA	FR, MT	[32]
	*A. oxyrinchus desotoi*	AC, RA	MT	[24]
	*A. oxyrinchus desotoi*	RA	MT	[219]–[221]
	*A. sinensis*	AC	FR, MT	[63]
	*A. sinensis*	AC	FR, MT	[64]
	*A. sinensis*	AC	FR, MT	[65]
	*A. stellatus*	AC	FR, MT	[58]
	*A. transmontanus*	AC	MT	[222]
	*A. transmontanus*	AC	FR	[223]
	*A. transmontanus*	AC	FR, MT	[78]
	*A. transmontanus*	AC, RA	FR, MT	[224]
	*A. transmontanus*	AC	FR	[141]
	*S. platorynchus*	RA	MT	[225]
**Large-scale migration (includes marine)**	*A. medirostris*	AC	FR	[35], [39], [40]
	*A. medirostris*	PAT	AG	[34]
	*A. oxyrinchus desotoi*	AC	FR	[226]
	*A. oxyrinchus desotoi*	AC, RA, PAT	MT	[29]
	*A. oxyrinchus desotoi*	AC, PAT	MT	[27]
	*A. oxyrinchus desotoi*	AC, RA, CART	MT	[227]
	*A. oxyrinchus desotoi*	AC, AR, CART	FR, MT	[26]
	*A. oxyrinchus desotoi*	AC	FR, MT	[228]
	*A. transmontanus*	AC	FR	[186]
**Movements and habitat use within estuaries**	*A. medirostris*	AC	FR	[79]
	*A. medirostris*	AC	MT	[229]
	*A. oxyrinchus*	AC	FR, MT	[80]
	*A. oxyrinchus desotoi*	AC	MT	[230]
	*A. sturio*	AC	MT	[52]
**Behaviour of cultured fish after release**	*A. naccarii*	AC	MT	[85]
	*A. oxyrinchus oxyrinchus*	AC	FR, MT	[53]
	*A. sturio*	AC	FR, MT	[53]
	*A. sturio*	AC	FR, MT	[54]
	*A. transmontanus*	AC	FR	[86]
	*S. albus*	AC	MT	[87]
	*S. albus*	RA	MT	[88]
**Passage through fish ladders**	*A. brevirostrum*	RA	FR	[89]
	*A. transmontanus*	CART	FR	[90]
**Habitat use and movements in lakes**	*A. brevirostrum*	RA	MT	[231]
	*A. fulvescens*	RA	MT	[232]
	*A. fulvescens*	AC	FR	[67]
**Identifying spawning habitat**	*A. brevirostrum*	AC	MT	[92]
	*S. platorynchus*	AC, AR	MT	[93]
**Dam operations (response)**	*A. sinensis*	AC	FR, MT	[233]
	*A. transmontanus*	AC, EMG	MT	[94]
**Survival (induced spawning)**	*A. oxyrinchus desotoi*	AC	MT	[234]
**Cultured fish survival**	*A. fulvescens*	AC	FR, MT	[235]
**Demographic parameters**	*A. oxyrinchus*	AC	FR, MT	[236]
**Spawning success**	*S. platorynchus*	AC, AR	MT	[81]
**Habitat evaluation above barrier**	*A. brevirostrum*	RA	MT	[91]
	*A. medirostris*	AC	FR, MT	[38]
**Basic life history**	*A. brevirostrum*	CART	FR, MT	[237]
**Freshwater habitat, movement**	*A. fulvescens*	RA	MT	[238]

**Tag Types**: AC  =  Acoustic; RA  =  Radio; AR  =  Archival; PAT  =  Pop-off Archival; CART  =  Combined Acoustic and Radio; EMG  =  Electromyogram.

**Tracking Method**: MT  =  Mobile Tracking; FR  =  Fixed Receivers; AG  =  Archival Geolocation.

The second most frequent use of electronic tags for sturgeon research has been to investigate large-scale migrations (e.g., movement of sturgeon among riverine, estuarine, and marine waters). These studies have used all types of telemetry tags and tracking methods, and most have used acoustic tags, or a combination of acoustic and other tag types. The study of [35] is notable for its use of cross-shelf arrays of receivers to document annual migrations of Green Sturgeon (individuals of which can cover more than 4000 kms per year). This was possible because the continental shelf off the west coast of North America is narrow, and a number of studies were underway using compatible tags and receivers, including the Census of Marine Life's Pacific Ocean Shelf Tracking (POST) program [78].

Movement and habitat-use studies in estuaries (*n* = 5) have relied on acoustic tags and mobile tracking, with the exception of a study of Green Sturgeon in Willapa Bay (Washington) and the Columbia River estuary [79] which used arrays of fixed receivers, and a study of Atlantic Sturgeon in the St. Lawrence River estuary that used mobile tracking and one fixed receiver [80].

The study by [81] used a combination of archival tags, radio tags, and sophisticated data analysis to determine if mature Shovelnose Sturgeon spawned successfully following tagging. The radio tags were used mainly to relocate individual sturgeon (whose state of gonadal development had been determined) bearing the digital storage tags that recorded temperature and depth. The analysis involved fitting a switching model to the depth histories of individual sturgeon; spawning was inferred by observing a period of reduced depth variability. Data provided by electronic tags, especially from archival tags, can be a rich source of information on behaviour that can be analysed with appropriate models that connect behavioural modes to the tag data [82]–[84].

Electronic tags have also been used to investigate the behaviour of cultured sturgeon following release [85]–[88], the behaviour of sturgeon near potential fish passage impediments [89], [90], experimental evaluations of habitat suitability [91], and to identify spawning habitat [92], [93]. Both acoustic and electromyogram (EMG) tags have been used to test the response of White Sturgeon to changes in dam operations [94]. EMG tags transmit radio pulses when muscles contract, allowing researchers to measure the physical activity of fish at finer scales than can be determined from tracking techniques that only provide coarse changes in position over time, or in flow fields where swimming speed in water may differ substantially from speed over ground.

**The importance of sex determination in telemetry studies** – Many biotelemetry studies have been conducted with juvenile and adult sturgeon without identification of sex and stage of maturity. In certain cases, such as identification of general habitat use or spatial distributions of a population, it may not seem imperative to know the sex of individuals. However in many cases, such as when trying to understand why habitat is chosen, or when and why migrations are initiated or ceased, identifying the sex and, in adult populations, the stage of maturity provides greater insight into the behaviour of the animal [24]. It often is stated in the literature that sex and stage of maturity were not determined to reduce stress; it is critical to reduce stress, but trained personnel can determine sex and stage of maturity with very little increased handling time during transmitter application, or blood plasma levels can be examined for retrospective analysis. A review of methods is provided here [95].

If transmitters are attached externally, ultrasonography or endoscopy may be used to determine sex and maturity [96]–[102]. The primary limitation of these techniques is that it is often difficult to differentiate immature females from males; in addition, the stage of maturity in males cannot be determined as size of testicular lobe does not always confer stage of maturity. Ultrasound and endoscopy are useful tools in the identification of ripe females. When transmitters are implanted surgically, a sterilized otoscope or pen light may be used to visually determine sex and stage of maturity. A larger incision than the incision for insertion of the tag is not required under these circumstances. However, as in the case with ultrasound and endoscopy, the stage of maturity in males cannot be determined visually, though a small biopsy may be collected for histological analysis and stage of maturity determination. Additional limitations of ultrasonography are the potentially high cost of equipment and the moderate level of training required in order to interpret sonograms.

Measurement of circulating sex steroids may be used less invasively to determine sex and stage of maturity [67], [103]–[107]. Under ideal circumstances, the misclassification rates of assigning fish to classes of sex and maturity (i.e., pre-vitellogenic female, pre-meiotic male, vitellogenic female, etc.) using plasma steroid concentrations would be determined for each species. Interestingly, when classification functions derived for White Sturgeon to predict sex and stage of maturity [104] were applied to a small number of Lake Sturgeon, comparable classification rates were found [106]. Special consideration for the use of this tool should be made in populations that may be exposed to environmental contaminants [107].

**Stress associated with capture and handling in telemetry studies** – Less is known about the neuroendocrine control of the stress response and roles of allostasis and hormesis in chondrosteans compared to teleosts [108]. However, the cortisol response has been described in several sturgeon species in response to a stressor [108]–[112], and cortisol has been identified as the primary glucocorticoid in Pallid Sturgeon [113]. Plasma cortisol concentrations (basal and stressed) in sturgeon vary by species [109], [113]; variation in plasma cortisol concentrations may also be influenced by time of day, age, size, season, temperature, and capture and sampling techniques [110], [112], [114], [115]. It is essential to reduce stress (i.e., air exposure, handling time, considering water temperature, etc.) during external attachment or surgical implantation of transmitters. Guidelines for the reduction of stress to fish following capture and during handling/sampling are provided in [95], [116].

### 2.2. Experimental Design for All Tagging Studies

In order to design a cost-effective and useful tagging program, researchers should compose an experimental design that considers sample size, the costs associated with tagging and recapture activities, and study duration [117]. For example, when making inferences about temporal movement patterns using archival tagging data, the use of representative data sets (covering appropriate spatial and temporal scales) with a large number of reconstructed migrations (ideally 100+) is recommended [118]. With the exception of telemetry studies (where data may be recovered remotely), consideration should be given to the potential number of tags that are expected to be recovered (based on the number of tags deployed and the level of subsequent sampling effort to recover tags) and reported. Low tag reporting rates can lead to low precision in estimates of rates of natural and fishing mortality [119]. Ideally, tagging and marking studies should aim to minimise tag loss and tagging-related mortality and be done in a way that the tag reporting rate is known or can be estimated [119] [122]. Researchers should consider the statistical power of a planned tagging experiment, and whether their sample size will provide adequate power to detect the effect size of interest [123] [124]. Further stratification by length, age, sex, or life-history stage may also be desirable [125]; in this case, the number of releases will need to be scaled up by the number of classes or life-history stages for which parameters will be estimated to achieve the desired level of precision and accuracy [126].

The spatial and temporal distributions of sampling/tag deployment also warrant consideration. Tagging and release in each area combined with availability of fishing/sampling effort by time in each area has been recommended to minimize bias and the standard error of movement rate estimates [126], [127]. The intensity of fishing/sampling effort and the underlying movement rates themselves will also affect the accuracy and precision of movement rate estimates; ideally more tags should be released in areas with low rates of originating and incoming movement [127]. In the context of exploitation rate estimation, tagged fish should be representative of the exploited population, such that each exploitable fish is equally likely to be tagged [128].

### 2.3. Data Analysis and Modelling

Information from marking and tagging experiments can aid the design of subsequent surveys and the interpretation of the survey data. This is particularly relevant to Caspian Sea sturgeons as total allowable catch estimates are based upon population assessments derived from trawl survey data. Spatially-structured mark-recapture models provide a suitable framework for estimation of rates of residency and movement using tagging data [125]–[127]. Tagging data also allow inference about population abundance and exploitation rates [128], providing that rates of tag loss and reporting are known or estimable [129], [130]. Electronic tags generate relatively new kinds of data, and methods for the analysis of these data are lagging behind the rapid development of the tags, and the increasing use of large release groups. In the early studies, when tags were more expensive and their utility unproven, typical studies tagged a handful of animals. In this case, data arising from each tag could be presented separately and in full. Newer studies have tagged hundreds or even many thousands of fish (e.g., studies of in-river migration in Pacific salmonids), requiring statistical analyses to estimate demographic parameters or infer behaviours.

Methods for analyzing the rich data streams from archival tags are relatively well-developed. Capture-recapture methods, such as the multi-strata robust design, would seem to be applicable to situations where sturgeon move among many different areas and experience different mortality rates according to their location or state (e.g., maturation status, sex); however, in order to achieve reliable outputs, several challenges must be solved. These include achieving large-enough sample sizes to reliably estimate the many parameters of such models, and dealing with the problem that detections of tagged sturgeon occur over the same time interval as the mortality process. Typical capture-recapture models assume that there is no mortality during the recapture process, which in some cases can be instantaneous.

The data gathered from tagging studies that use simple (internal or external) tags and/or telemetry can potentially provide information on a number of life-history characteristics that are necessary to model population dynamics (such as growth, rate of natural mortality, and spawning periodicity). Using life-history traits to understand how populations may respond to perturbations such as harvest has been advocated as a useful approach to predicting extinction vulnerability and formulating management strategies [131]–[133]. Population dynamics models can be applied to gain an improved understanding of the status of sturgeon populations and their response to exploitation (historical and/or projected). The majority of population dynamics models for sturgeon species to date can be classified as simulation models, which can be applied to evaluate the sensitivity of population productivity and growth rates to changes in the survival rates of different life stages, or to estimate maximum sustainable yield harvest rates [134]–[137]. Where a time series of historical catches is available, population dynamics models can be used to reconstruct unfished population abundance over time to estimate depletion (the ratio of current to unfished abundance or biomass) [138]–[140].

### 2.4. Genetics

Applying genetic techniques allows a researcher to understand the species that are present in a given area (i.e. native, non-native, hybrid, or aquaculture origin) and the movements of sturgeons on multiple spatial scales and through evolutionary time [see references in this section below]. An important question that can be addressed using genetics is that of where an individual animal reproduces. As a first step in answering this question, reproductively isolated populations of a sturgeon species must be identified through genetic analysis. By knowing the number of isolated populations, appropriate conservation schemes can be created that preserve the evolutionary and ecological potential of a species. The protection of multiple populations can serve to buffer against extinction due to environmental change. Many sturgeons are thought to return to their natal river to reproduce and it is this behaviour that creates genetic structure and separate populations. It is unlikely, on the timescale of interest to managers, that animals from one river system would replace those in another if an individual population were to go extinct; thus, it is important to minimize individual population extinction since translocation of individuals from other populations should only be used as a last resort. A related point is that of identifying the presence of non-native species or hybrids in an area as an important step in conservation planning.

While information on the location of reproduction can be obtained through tagging studies, this approach does not answer the question of whether the animal effectively breeds in an area. Evidence of spawning can be gathered through biochemical means (e.g. measuring hormone levels or gonadal development), the deployment and monitoring of egg-mats or larval traps, or specifically designed electronic tagging studies, but only genetics can pinpoint whether the individual contributes to the next generation. In addition, it may be easier and less costly to use genetics to track breeding. As described below, once the overall genetic structure of a species is characterized, it can be fairly straightforward to identify the population origin of any individual at any location. Combining tagging and genetics can further provide a more comprehensive picture of population structure as defined by present and historic habitat characteristics [141].

Some of the fundamental questions that can be addressed using genetics include:

- Which species or species hybrids exist and reproduce in a given area (especially areas that may have been influenced by captive population release, both intentional and unintentional)?
- How many populations exist within a species? Where do individuals from these populations breed and feed?
- Does a specific area in the ocean or in an estuary include individuals from a single population or multiple populations and, if the latter, which ones?
- Do two or more populations exchange individuals?

Ideally, a genetics study will begin with comprehensive sampling of all potential spawning populations. For some sturgeons (e.g. Shovelnose, Pallid, White, possibly Russian), multiple spawning populations may exist within a single river system. Population differentiation can result from differences in timing or geographic location of spawning and can occur in the absence of any physical barriers separating populations. The samples taken from each population should ideally have corresponding information about the relative age of the animals (larvae, juvenile, adult) and the spawning stage of an adult. Information on life stage of the sample becomes especially important when sampling is conducted in lower reaches of the river and in estuaries because individuals can congregate in estuaries and coastal environments that are not in close proximity to their natal river of origin. When species or hybrid identification is a concern, sampling multiple life-stages can yield insight on whether non-native species or hybrids are effectively breeding in an area.

Sequencing of mitochondrial DNA (mtDNA) is one of the easiest genetic techniques to use for identifying the species present in an area (including non-natives) and studying movement, distribution and population structure and has been extensively applied e.g. [50], [142], [143]. Examples of the use of mtDNA include characterizing the presence of non-native Siberian Sturgeon haplotypes in Russian Sturgeon in the Caspian Sea [144]–[147] and the presence of non-native sturgeons in rivers in Greece [148]. One or several rapidly evolving gene segments (e.g. control region, cytochrome b) are usually studied, but sequencing mitochondrial genomes is now common. The utility of the latter is debatable given that most of the variation exists within the control region segment. The mtDNA cytochrome b and control region are also useful in differentiating among species [149]–[151], so researchers working in areas where multiple sturgeon species coexist can conduct species identification with mtDNA. Methods such as sequencing or Restriction Fragment Length Polymorphism (RFLP) analysis can be used to study population structure. The caveat in using mtDNA is that it tracks only the maternal lineage; this is important where species hybridize or where hybrids may be present and/or sex-specific dispersal may occur.

With respect to delineating population structure, one of the best examples of the utility of mtDNA comes from the east coast of North America. The Atlantic Sturgeon has been particularly well studied and this work has shown fine-scale population structure and spawning site fidelity [142]. The data have been used to develop schemes of population-level protection under the US Endangered Species Act and attributing population origin to individuals captured in the ocean. Given that tagging studies are revealing large-scale oceanic movements at significant distances from natal rivers, this is particularly important because fisheries operating at considerable distances from a natal river could be impacting that population. For example, genetic samples taken from Atlantic Sturgeon captured off of the New York Bight (north eastern US waters) indicated that some of the sturgeon sampled were from breeding populations several hundred kilometres to the south in south-eastern US rivers [152].

Unlike mtDNA, microsatellites are biparentally inherited and genetic studies using these markers would not be biased by sex-specific differences in movements. In addition, microsatellites are highly polymorphic and may reveal population structure on a finer scale than mtDNA. There is less ease in applying this technique across species and species-specific marker development is sometimes necessary. Complications can arise in using microsatellites for polyploid sturgeons (i.e., species with greater than two copies of each chromosome), as some species may possess eight or twelve chromosome copies, however, the use of microsatellites to study populations can be achieved if Mendelian patterns of inheritance of microsatellites are found [153], [154]. General concerns about microsatellites have been raised by many authors [155]. Adriatic, Atlantic, Green, Lake, and White Sturgeon have been the most-studied in terms of the application of this marker; Caspian Sea and Chinese species are also becoming the subject of microsatellite investigations. Microsatellites are being used to understand and monitor hybridization in Scaphirhynchus species (with limited success when successive back-crossing occurs [13]), and hybridization between exotic and endemic species in the Danube River [156].

Microsatellites have been particularly useful in characterizing the marine distribution of Green Sturgeon and these studies, along with those on non-marine oriented sturgeons (White, Shortnose) [153], [154] have demonstrated application in polyploid sturgeons. Within the Green Sturgeon range, two distinct population segments (DPS) have been identified using genetics, corresponding to northern and southern spawning locations [47]. Individuals from the two DPSs often mix in the estuarine habitats of both natal and non-natal river systems. A further application of microsatellites in Green Sturgeon has been the generation river-specific abundance estimates [157]. Microsatellites have been used to characterize the population structure of Atlantic Sturgeon, to design a scheme of DPSs, and to characterize mixed-population fisheries [158]. Regarding the latter, microsatellite analysis revealed that Atlantic Sturgeon captured in US waters off of Virginia and North Carolina originated from populations as far north as Canada and were composed to a large extent of animals originating in the Hudson and Delaware rivers [159].

Many studies use both mtDNA and microsatellite markers. One interesting application, which also used four single-nucleotide polymorphisms (SNPs), is the study of the historical distribution of European and Atlantic Sturgeon. Study of archived and historic specimens revealed that Atlantic Sturgeon colonized an area in the Baltic Sea that was formerly thought to only be inhabited by European Sturgeon [160]. This work not only revised previous assumptions about species distributions, but also allowed restoration efforts to move forward in areas of Europe where sturgeons have been extirpated. Similar applications are ongoing to understand the former distribution of these sturgeons throughout Europe. Another important application has been to detect whether pure or hybrid strains of sturgeons are present in an area, thereby guiding captive breeding initiatives [161].

Emerging approaches include single-copy nuclear genes and SNPs [155], [162]. The application of these approaches to sturgeon has been limited because of the complications associated with polyploidy. Experimentation is ongoing with development of SNPs in Lake Sturgeon, White Sturgeon, some species of Eurasian and US sturgeons. Once developed, SNPs will offer additional power in determining population structure and movement.

There are many different analytical tools in the field of population genetics that can be applied. Measures of genetic differentiation such as genetic distance, F-statistics, analysis of molecular variance (AMOVA), and exact tests can be employed to identify distinct sturgeon populations and measure gene flow between them. These methods are most useful when samples can be obtained from spawning adults or newly hatched larvae in a natal river, where a researcher can be assured that the individuals sampled belong to a particular spawning population. When sampling is conducted in a region of potential mixing between populations (lower river, lake, estuary, ocean, or sea), the origin of each individual examined is unknown and other methods are necessary to examine population structure. Population assignment testing can be used with microsatellite or SNP markers to identify the population of origin of individual sturgeon in an area of potential mixing. There are several types of population assignment tests but they all exploit differences in allele frequencies between populations to assign individuals to their natal population. The software program STRUCTURE [163] may be particularly applicable to sturgeon studies as it can accommodate polyploid microsatellite data. In addition to identifying the populations contributing to a mixed population [47], population assignment tests can be used to evaluate individual dispersal behaviour. The assignment program GENECLASS2 [164] was used to identify two migrant sturgeon that originated from other river systems in the remnant Lake Sturgeon population in the White River, Indiana [165]. Finally, population assignment tests might be used to study population structure at varying hierarchical scales; STRUCTURE was used to examine groups of related Lake Sturgeon populations in the Great Lakes basin [166].

### 2.5. Microchemistry

Differences in trace elemental profiles between habitats or bodies of water can be exploited to learn more about the migratory behaviour of fishes. Gradients in elements such as Sr, Ba, Ca, Mg, S, and B exist in regions of different salinities, temperatures, and bedrock influences [167]. These elements are incorporated in minute quantities into the calcified structures of fishes, such as otoliths, fin rays or spines, bones, and scales [167]. Differences in the presence or concentrations of trace elements among aquatic habitats create elemental “fingerprints” on calcified structures that can be used to determine where a particular individual originated [168]–[170]. This information may be used to detect population structure, particularly in species where individuals from different populations mix during non-reproductive times, as has been done with some shell-forming invertebrates [171].

Otoliths are used most often in microchemical analyses of fishes since, unlike scales or skeletal bones, there is no potential for resorption or remodelling of this structure [172]. Although fin rays have the potential for remodelling, several researchers have shown the stability of elemental signatures in fin rays over time, suggesting they are stable structures appropriate for use in microchemical analyses [60], [170], [173].

One advantage of otoliths and fin rays is that changes in elemental composition between regions of incremental growth may be used to reconstruct patterns of movement in these fishes over time [174]. Most work to date has focused on otoliths, although conducting microchemical analyses on fin rays holds much promise for long-lived species such as sturgeons. Microchemical analysis of non-lethally collected fin rays has the potential to reveal age or stage-specific movement behaviour or habitat preferences in threatened or endangered sturgeon species, where otolith collection is undesirable.

#### 2.5.1. Application to Sturgeon Movement

Applications of microchemical techniques to understanding sturgeon movements have been limited to this point, focused primarily on examining changes in marine and freshwater habitat use over the lifetime of an individual. In a study that confirmed the utility of fin rays for examining sturgeon movements [173], no changes in chemical composition due to bone remodelling or fin ray resorption over time were detected in individual White Sturgeon. This study also showed a significant correlation in the deposition of many trace elements between fin rays within individuals over time [173], suggesting that deposition occurs in a predictable way. Since then, several researchers have exploited predictable differences in Sr:Ca ratios between freshwater and marine environments to examine migration of various life stages of sturgeon from freshwater to marine habitats.

Approximately 10% of lower Fraser River White Sturgeon subadults between ages 1–15 showed Sr:Ca ratios that were consistent with migration into the marine environment, although these movements did not appear to be seasonal or periodic [175]. Intermediate concentrations of Sr:Ca ratios in fin rays of 58% of Fraser River adult White Sturgeon examined were suggestive of estuary use between ages 1–15 [175]. Sr:Ca ratios in both otoliths and fin rays were used to detect freshwater-to-saline migrations of subadult and adult Russian Sturgeon [176], [177]. Some individuals were characterized by a single movement into the Caspian Sea (from freshwater), while other adults had patterns consistent with diadromous movements between freshwater and seawater [176], [177]. The ratios of Sr:Ca, Ba:Ca, and Sr:Ba were used to evaluate the age of marine entry of subadult Green Sturgeon [178]; this study also conducted *ex situ* experiments to evaluate whether elemental deposition of Sr, Ba, and Ca in Green Sturgeon fin rays was proportional to environmental concentrations of these elements in freshwater and seawater environments. It was confirmed that ambient ratios of elements in freshwater and saltwater were nearly identical (Sr:Ca) or proportional (Ba:Ca; Sr:Ba) to the ratios found in fin rays of individuals held in freshwater or saltwater, respectively [178]. Wild Green Sturgeon subadults enter brackish estuary habitat between 0.5 and 1.5 years of age, and make their first migration into marine habitat between 2.5–3.5 years of age [178]. The ratios of both Ba:Ca and Sr:Ca ratios aided in interpretation of more complex environmental histories involving transitions between freshwater, brackish estuary, and marine habitats [178]. Although not the goal of these movement studies, some local differences in elemental concentrations [173], [178] suggested the potential for elemental “fingerprinting” in population composition analysis for sturgeon.

#### 2.5.2. Future Research

Microchemistry techniques might be applied to resolve many questions regarding sturgeon movements and habitat use. Although there are some uncertainties associated with deriving age estimates from sturgeon fin rays [179], [180], the ability to examine habitat preferences and movement patterns of approximate age classes will be very useful in enhancing our understanding of sturgeon life history, particularly for those species with access to numerous and complex habitat types. Of particular interest would be the identification of important nursery or rearing habitats for early life stages of sturgeons, as this is unknown for many species. One possible use of elemental “fingerprinting” would be in examining spawning site fidelity in sturgeon. Although homing to natal spawning grounds has been assumed for many species, the long generation times of wild sturgeon have, to date, precluded direct confirmation through tagging studies. Population genetic data for several species [142], [166], [181] show genetic structure between spawning populations, which suggests that sturgeon do exhibit homing behaviour. However, elemental profiles in the core of fin rays of spawning adults might be compared to ambient elemental profiles in spawning habitats, which could confirm the hypothesis that the adults returning to spawn in a particular stream or river originated from there as well. Before these types of analyses are attempted, however, experimental work must establish that larval residency time in natal rivers is long enough for the incorporation of ambient elements in detectable concentrations. Also, it will be important to establish whether trace elements detected in otoliths and fin rays are consistently represented proportionally to ambient trace elements. A study of marine larval fishes [169] found that only one of 12 elements examined showed a similar correlation between ambient concentration and otolith deposition in an open-sea environment. The reported [178] proportional relationship between fin ray trace element concentration in Green Sturgeon and ambient water concentration might have been due to the coarse scale (freshwater vs. saltwater) at which environmental differences were examined.

Microchemical techniques have the potential to provide a great deal of information about sturgeon movement and life history. However, interpretation of elemental profiles in fish bony structures is dependent on an accurate understanding of the factors that influence element deposition. Additional work to ascertain the influence of water temperature, salinity, and the interaction between these parameters on element deposition in sturgeon otoliths and fin rays must be conducted to avoid misinterpretation of elemental profiles [182]–[184]. In addition, the use of laser ablation-inductively coupled plasma mass spectrometry (LA-ICPMS) techniques on otoliths from other species have shown ontogenetic changes in element deposition [185]; these changes could be quite confounding when reconstructing historical movements and habitat preferences of sturgeon and other long-lived fishes. Diet, too, may affect element deposition in otoliths and fin rays. We also must determine how residency time in a particular habitat affects element deposition [182]. Sturgeon are capable of fairly rapid movements between habitat types [79], [186], [187] and it is uncertain if these movements would be represented accurately in otolith or pectoral fin ray elemental “fingerprints.” Also, it will be important to characterize the rate at which elemental profiles within particular locations vary, as rapid rates of naturally or anthropogenically induced environmental change may reduce our ability to accurately reconstruct the movement of individuals over longer timeframes using otoliths and fin rays [188]. These uncertainties should be addressed prior to a broad application of microchemical techniques toward addressing questions regarding sturgeon movement and habitat preferences.

### 2.6. Observational Technologies

Sturgeon can occupy marine environments or large, deep and typically turbid river systems where direct visual observation of behaviour, movement, or habitat use is difficult and often impossible. Consequently, until recently, what little was known about these attributes of sturgeon ecology were based on anecdotal observations or inferences drawn from capture and tagging studies. In the last two decades, substantial advances in remote sensing technologies have provided researchers with a greater variety of tools that allow direct observation of sturgeon behaviour and habitat use. These technologies can generally be grouped into two categories: those that use underwater cameras to provide light-based images and those that rely on sound waves to produce sonic-based images. Each of these observational categories and their applicability for use in studying sturgeon behaviour and habitat use is discussed below. Although most of the applications encountered in the reviewed literature were used for the study of sturgeon in freshwater systems, in many instances they also have applicability for the study of sturgeon and their habitats in marine and estuarine environments.

#### 2.6.1. Underwater Photography

The use of underwater photography as a tool to examine sturgeon behaviour has only recently begun to be explored. Advantages of using underwater cameras are the ability to directly observe fish in their natural habitats and for some sturgeon species, the absence of any apparent avoidance behaviour of underwater cameras and lights. Disadvantages include limited use in aquatic environments with low water clarity and difficulties in long-term monitoring of fish that are engaged in active feeding or large-scale movements.

Obtaining underwater footage has been facilitated in recent years through the development of small submersible Remote Operated Vehicles (ROVs) and technological advances in low-light digital and video cameras. ROVs have been used in freshwater applications to examine the real-time effects of hydroelectric plant operations on sturgeon feeding behavior [189]. Underwater videography using ROVs has been employed for several years in the upper Columbia River in Canada to document behavior and habitat use by wild White Sturgeon adults and by hatchery-reared and released juveniles [5] as well as to document unusually large (approximately 60,000 fish) aggregations of White Sturgeon in the stilling basin below the spillways at Bonneville Dam (http://videos.oregonlive.com/oregonian/2008/05/sturgeon_ball.html). An ROV also was used to identify critical overwintering habitats for the endangered White Sturgeon in the Nechako River, a tributary to the Fraser River in British Columbia, Canada [190].

Underwater video camera systems have been used to study overwintering habitats of Shortnose Sturgeon in the upper Kennebecasis River, New Brunswick, Canada [191], characterize Lake Sturgeon spawning and substrate preference in the Big Manistee River in Michigan [192], and identify White Sturgeon spawning and early life-stage rearing substrates in the upper Columbia River in Canada [193]. White Sturgeon use of tailrace areas below existing hydroelectric dams in the Columbia River (Canada) was also examined using fixed video cameras and the information generated led to a plan to protect sturgeon during tailrace excavation [194]. White Sturgeon mortality at the Brilliant Expansion power plant was reduced after video monitoring of the powerplant draft tubes and outlets illustrated behavioural responses to reduced flow [195]. Cameras were used to provide real-time data on sturgeon presence during short duration forced outages of the powerplant; video footage recorded after the shut down were reviewed by the dam operators to assess if sturgeon have entered the draft tubes. Depending on the results, different start-up protocols were implemented to reduce risks to sturgeon.

#### 2.6.2. Hydroacoustic Technologies

Over the last three decades, active hydroacoustic techniques have developed and proven to be a relatively easily applied method of unobtrusively evaluating fish populations in freshwater and marine environments [196]. The principles of hydroacoustic assessment of fish are provided in [197] and [198]. Hydroacoustic techniques have a very high sampling power and do not affect fish health, behaviour, or the environment being monitored and have been successfully applied to a variety of fisheries evaluations, including both mobile and stationary assessments of aquatic systems.

Mobile survey hydroacoustic techniques are generally conducted from a boat, traversing predetermined transects in a body of water, and sampling fish and bottom characteristics [196]. Sampled fish produce characteristic acoustic signals that can be processed using specialized software to produce estimates of fish density, abundance, behaviour, and size distribution. Sonars and sounders have been developed that can be used to characterize sea, lake and river bottoms and profiles of the upper layers of the ocean bottom. Advanced substrate classification analysis can be achieved using calibrated (scientific) echosounders and parametric or fuzzy-logic analysis of the acoustic data. Side-scan sonars can be used to derive detailed maps of the topography of an area by moving the sonar across and just above the bottom. Low frequency sonars have been used for continental shelf wide surveys while high frequency sonars are used for more detailed surveys of smaller areas. Various synthetic aperture sonars (SAS), which combine a number of acoustic pings to form an image with much higher resolution than conventional sonars, are under active development (http://www.hydro-international.com/issues/articles/id920- Synthetic_Aperture_Sonar_Challenges.html). This technology has become commercially viable and the technique is well suited for towed or remotely operated underwater vehicles. SAS is expected to replace traditional side-scan sonars for many applications in the future.

All acoustic systems have some sampling limitations with respect to their ability to resolve targets very close to boundaries, such as the bottom. Sturgeon are primarily benthic oriented and often in close proximity to the bottom. In addition, research describing sturgeon target strength, or the amount of acoustic energy reflected from the fish, is limited. To be effectively detected, a fish must return target strength values greater than the surrounding background noise levels. The primary reflecting structure in most fish is the swim bladder, although bones, scutes and other body structures do provide some contribution [199]. The sturgeon swim bladder is the primary acoustic reflecting structure and is located just below the spine, some distance from the ventral surface of the fish. This may aid in detecting these fish on the bottom with a down-looking acoustic system, as there is some inherent separation between the upper surface of the bladder and the bottom itself due to fish morphology [196].

**Split Beam** – The main advantages of split-beam over other hydroacoustic techniques are improvements in location within the acoustic beam and in minimized susceptibility to noise [200]. Given identical levels of bias in angular resolution, the split-beam system can locate fish within the beam with much greater resolution than single-beam, dual-beam, or sidescan systems [201], [202]. The three-dimensional location of each fish is known for each ping (i.e., each ensonification). This improved spatial resolution results in improved target strength estimates. More accurate target strength estimates allow more accurate spatial expansions, resulting in more accurate estimates of fish abundance and/or biomass. With split-beam target tracking, individually measured echoes may or may not be retained, depending on selection criteria that discriminates fish echoes from other echoes. Selected echoes are tracked to group all echoes from one individual fish. Mean target strength is calculated from the group of echoes from one fish, based on individual-echo target strength measurements made using the split-beam method. As with the other techniques, the signal can be echo integrated, to provide biomass estimates, if desired.

Split-beam hydroacoustics was used to detect Shortnose Sturgeon in the Delaware River [196]. The study was conducted by measuring Shortnose Sturgeon target strength (using net-captured fish) and the range from the bottom at which sturgeon could be acoustically resolved. The authors concluded that Shortnose Sturgeon could be readily detected by a split-beam hydroacoustic system using a combination of attributes (target strength, position relative to the bottom, and echo envelope shape). The demersal distribution of Shortnose Sturgeon is well established and also appeared to be a useful metric for distinguishing these fish from other species.

Fixed-location split-beam sonar technology was used successfully to identify adult Lake Sturgeon as they moved upstream and downstream for spawning in the Sturgeon River, Michigan [203]. Data collected included direction of movement, swimming speed, range from transducer, time and date of passage, and target strength. The Lake Sturgeon spawning population size was estimated and results showed that spilt-beam sonar can be applied to spawning assessments, without the stress of actually handling the large, pre-spawning fish.

**Side Scan** – Trawling and side scan sonar analysis was used to document an area of consistently high Lake Sturgeon density in Lake St. Clair near the St. Clair River delta [204]. Side scan sonar was used to estimate the abundance of Lake Sturgeon in a 255-ha section of the lake and the data were used to enhance protection and habitat restoration efforts for Lake Sturgeon in this and other Great Lakes connecting waters.

On the lower Missouri River, side-scan sonar data were collected in areas that contained suitable habitats for both Shovelnose Sturgeon and Pallid Sturgeon [205]. Hydroacoustic data sets were collected at the reach scale (mean reach length, 2.4 kilometres) to include the immediate vicinity of a targeted sturgeon location as well as the full range of adjacent habitats. The images obtained were useful for visualizing channel substrate and detecting the presence of adult sturgeon.

Researchers in Virginia used side-scan sonar to characterize the benthic habitats of Atlantic Sturgeon in the James River and identify habitat attributes that may be required to sustain viable sturgeon populations (http://www.thsoa.org/hy09/0512P_02.pdf). Areas of high-frequency sturgeon occurrence (as determined through concurrent telemetry studies) were targeted using a side-scan sonar system. Numerous habitat features were identified and the data will be integrated with tracking data and habitat imagery to identify essential Atlantic Sturgeon habitats.

**Broadband** – Experiments were conducted in the tidal Delaware River to determine if Shortnose Sturgeon could be detected by broadband sonar and, if so, to develop classifiers that could differentiate Shortnose Sturgeon from co-occurring fish species [206]. The false-positive rate of incorrect identification of a Sturgeon was 16.5%. Notwithstanding this potential problem, the authors concluded that the results of this preliminary study were promising, and further investigations to improve classifier performance were warranted.

To assist in identifying potential Lake Sturgeon habitat in the lower Bad River complex, a digital sonar system combined with a global positioning system was used to provide georeferenced data, and specialized sonar, bottom typing, GIS and statistical software to acoustically map bottom substrate types, locations, and bathymetry [207]. Ground-truth data were developed from both petite Ponar bottom samples and associated acoustic data which were processed with bottom-typing software; these data were used to produce substrate models and maps.

**Fish finders** – Many commercial and recreational sturgeon anglers use high quality hydroacoustic gear or “fish finders” to locate sturgeon in estuarine and marine environments along the east and west coast of North America. For sturgeon research, a fish finder with the following features is best: multiple zoom settings, bottom lock, and split-screen option (http://www.nwfish.com/Sturgeon/fish_finders_101.htm). Units that allow setting the window size for a specific number of feet while in bottom track will provide higher signal resolution that will show any irregularities on the bottom as well as fish holding right on the bottom. Sturgeon that are suspended some distance above the bottom show up quite well on most high-resolution fish finders (http://www.nwfish.com/images/graphics/ffshotsturg1.jpg). Sturgeon holding tight to a hard bottom are difficult to identify and are usually represented by a “spike” or “bump.” (http://www.nwfish.com/images/graphics/ffshotsturg2%20.jpg).

**DIDSON^TM^** – In large rivers, a common approach for estimating population size of anadromous fish is to count upstream-migrating fish at a fixed site, using split-beam hydroacoustic equipment. A disadvantage of split-beam sonar in this application is that it generally does not provide sufficient information to allow species identification. A newer technology that can be used to count upstream migrants is the DIDSON^TM^ (dual-frequency identification sonar), a high-definition imaging sonar that provides near-video-quality images. When used at a range of 5–10 m, video files clearly show body shape and swimming behaviour of individual fish. Split-beam gear provides more precise information about fish position, but DIDSON^TM^ data are much easier to interpret, can be used to identify sturgeon to genus, and allow for on-screen measuring of fish lengths. Initial field trials showed potential for utilizing these technologies to determine habitat, identify sturgeon, and estimate densities (http://cars.desu.edu/aqua-sci/Abstracts/LB_et_al_acoustic.pdf). DIDSON^TM^ has also been used to document White Sturgeon presence and activity in the vicinity of power plant outlets [208].

Restoration and management of the lower Missouri River to support recovery of the endangered Pallid Sturgeon required quantifying habitats to isolate specific habitats that may present bottlenecks to reproduction and survival [209]. The approach taken involved intensive reach-scale hydroacoustic mapping using a suite of multi-beam bathymetry, Acoustic Doppler Current Profiler, high-resolution side scan sonar, and DIDSON^TM^ imagery, combined with intensive telemetric tracking. This approach provided measures of habitat availability and selection variables at sub-meter to bedform scales, commensurate with the scale at which fish occupied these habitats. The DIDSON^TM^ imagery indicated that during spawning, sturgeon occupy the lee slopes of dunes facing upstream (presumably to minimize energy expenditure) but episodically move out of dune fields and into deep, fast water over coarse substrate (presumably to release eggs and milt). This multi-scale, multi-instrument remote-sensing approach was essential for improving understanding of the linkages between life stages of a rare fish and its environment.

#### 2.6.3. Future Research

At present, observational techniques for sturgeon research have primarily been used to provide information on sturgeon behaviour and habitat use. These techniques enable researchers to directly observe sturgeon in their natural environment in a manner that does not influence their behaviour and have substantially increased our knowledge of how sturgeons interact with each other and their environment. Most importantly, these techniques have identified critical sturgeon habitats that in turn have resulted in the protection of those habitats through direct management regulations. As these observational techniques are developed further, their potential uses will continue to expand. Direct observation of sturgeon behaviour at dams and existing fish passage facilities, either through video or sonic imagery, can be used to provide information that may lead to the development of appropriate passage facilities for anadromous sturgeon. As sonar techniques are refined and new algorithms to process and interpret digital signals are developed, these techniques have the potential to be used to identify individuals and provide assessments of sturgeon distribution and abundance over large areas.

## Results and Discussion

The marine life history and distribution of many sturgeons remains a mystery. However, new technologies developed over the past two decades have greatly increased our knowledge base. Refined methods of analyzing DNA provide fine-scale information about genetic structure at the river basin or even sub-basin level. Improvements in telemetry equipment and observational techniques have allowed researchers to identify and characterize key habitats, from spawning sites at the upstream extent of migration to overwintering sites and migratory paths in coastal ocean waters. Telemetry and tagging studies have also shown that estuarine and marine sites used for foraging or overwintering may contain sturgeon from multiple populations. Tagging, genetic, and observational studies have revealed that sturgeon can travel considerable distances from their natal rivers, but that they generally inhabit coastal, shelf areas during their migrations. Also, individuals generally seem to return to natal rivers for reproduction. Taken as a whole, this information shows that sturgeons have complex life-history strategies that are reliant on specific habitats for different life stages. As such, fully protecting a given species requires a comprehensive approach that includes research and conservation efforts in riverine and marine environments.

Yet even with these technological advancements, there are few sturgeon species for which we have a fairly comprehensive understanding of respective life histories, particularly in the marine environment. For the 16 marine-oriented sturgeons, only two (Green and Gulf Sturgeon) have been well studied with respect to their marine distribution (see: Table 1; section 1.2), and even these species would benefit from additional study of the movements and habitat use of young juveniles during their initial years in marine environments. Current studies of Atlantic Sturgeon in North America are now providing an understanding of the marine life history of this species. For the remaining species, many of which are classified as Critically Endangered and some of which are still subject to commercial and recreational fishing, there is a pressing need to apply many of the tools discussed here. As illustrated in Table 2, only two species of sturgeon (Adriatic and European) outside of North America have benefited from electronic tagging studies and our review has indicated few other studies where other techniques have been applied to non-North American taxa. European and Asian species are clearly in need of studies that utilize the techniques presented. Thus, while we have numerous tools in our management toolbox, we are lagging in our application of these tools to study most sturgeon species.

The research tools and techniques reviewed can be used individually or in combination. In Table 3 we present a suite of common research questions (research focus) and rank each of the core research techniques in the context of achieving/producing the desired outcomes. As illustrated for many species, tagging, telemetry, genetics and sometimes observational techniques are often used in combination. Tagging and genetics are complementary tools for characterizing movement on an immediate and fine ecological scale (tagging) as well as an evolutionary scale (genetics). Tagging can also include multiple methods, with simple internal or external tags combined with electronic tags. Population dynamics models may help to direct both core study design and specific elements such as the number of tags deployed, the level of sampling effort, etc. Microchemical techniques may be used to complement movement studies and to add information on contaminants; however, the authors recommend that additional background research may be needed to correctly apply and interpret microchemistry results, and additional work is required to validate some aspects of the approach. Observational techniques are most useful in characterizing habitat uses within marine or freshwater environments, behaviour, and small scale movements, although recent advances have shown promise for use in assessing abundance and distribution as well.

**Table 3:** Schematic of techniques used to study different aspects of the biology of sturgeons.

	RESEARCH TECHNIQUE				
RESEARCH FOCUS	Genetics	External Tags or PIT Tags	Electronic Tags	Microchemistry*	Observational
Marine distribution	3	2	1	4	
Population structure and identification	1	3	2	3*	
Habitat characterization		4	2	2	1
In-river distribution		2	1	4	3
Population abundance/modelling	2	1	3		3
Response to disturbance (e.g. dam or dredging operation)		3	2		1
Life-history characterization (e.g. spawning periodicity, age at maturity, growth)		2	1		

* Microchemistry techniques, while not currently applied on a broad level to study population structure and identification, are building in popularity and applicability and may well rank alongside genetic and electronic.

tagging in the near future.

Research techniques are ranked 1–4 in order of use/utility by sturgeon researchers, with a rank of 1 being highest in terms of applicability. Techniques assigned the same number are of equal utility.

Tagging, telemetry studies, and photographic imagery can be useful ways to engage the public in research and raise awareness about the conservation status of sturgeons. Stewardship-based population monitoring and assessment programs that train individuals to inspect captured fish for the presence of a tag, apply tags to untagged fish, and collect basic information (fish length, girth, location, date) have been successful [75]. Telemetry studies, particularly those using comparatively expensive satellite tags, can be accomplished through individual or corporate sponsorship of a tag (and an individual fish); subsequent movement data generated can be shared with the sponsoring partner and with the public through a dedicated website. Imagery of sturgeon behaviour in the wild is a powerful visual tool that can be used to enhance public outreach programs and galvanize public support for population and habitat conservation, protection, and recovery. Elements of stewardship and public outreach programs can also be incorporated into many areas of school-based educational curriculum, including ecology, biology, natural history, environmental studies, and social studies: http://hsbc.frasersturgeon.com/.

Data collected by the methods described here can provide useful insights into the life history of sturgeon populations, and enable management and conservation. For example, information regarding temporal and spatial movements, migrations, and periodic residency, coupled with a general knowledge of life history events such as spawning and in-river overwintering, could provide a high level of confidence regarding the location and extent of proposed habitat protection for population conservation and recovery. Genetic data on population structure is essential for identifying particularly vulnerable or endangered populations and setting appropriate management actions to conserve them. Gaining an understanding of life-stage-specific movement patterns and spatial distribution is also required to properly assess the impacts of anthropogenic activities such as fishing, dredging, and gravel extraction and to specify management actions accordingly.

All of the techniques discussed require a certain amount of specialized training. Protocols for proper handling, short-term holding, tag applications, and tissue sampling have been produced for some species [210]; many of these established protocols are transferable to other species of sturgeon. Given the widespread use of many techniques, assistance can be readily obtained via contact with experienced researchers. Collaborative research is likely the best approach given that many techniques require up-front purchasing of expensive equipment and a steep learning curve. We hope that this review sparks interest and enthusiasm amongst researchers, especially those outside of North America, and provides some necessary tools and references for forming new studies.
